# Impact of the Hepatitis B Immunization Strategy Adopted in Italy from 1991: The Results of a Seroprevalence Study on the Adult Population of Florence, Italy

**DOI:** 10.3390/pathogens14040362

**Published:** 2025-04-07

**Authors:** Sara Boccalini, Beatrice Zanella, Marco Del Riccio, Benedetta Bonito, Massimiliano Alberto Biamonte, Mario Bruschi, Giulia Ionita, Diana Paolini, Maddalena Innocenti, Lorenzo Baggiani, Monica Della Fonte, Giovanna Mereu, Paolo Bonanni, Working Group, Angela Bechini

**Affiliations:** 1 Department of Health Sciences, University of Florence, 50134 Florence, Italymarco.delriccio@unifi.it (M.D.R.);; 2 Medical Specialization School of Hygiene and Preventive Medicine, University of Florence, 50134 Florence, Italy; 3 AUSL Toscana Centro, 50122 Florence, Italy

**Keywords:** ELISA, vaccination, serological marker, epidemiology, predictor, HBV, adult, acute infection, chronic infection

## Abstract

Italy was one of the first countries to implement a hepatitis B (HBV) immunization strategy in 1991; since its introduction, the epidemiology of this disease has significantly changed. The aim of this retrospective study was to assess the seroprevalence of three HBV markers (anti-HBs, anti-HBc, and HBsAg) and describe the acquired immunity in a representative sample of the adult general population in the province of Florence (Italy) between April 2018 and December 2019. We conducted an enzyme-linked immunosorbent assay on 430 serum samples collected from the adult general population to quantify anti-HBs titers and assess the presence of anti-HBc and HBsAg. For the interpretation of hepatitis B serologic results, we referred to the US CDC guidelines. We conducted two multivariate logistic regression analyses (applied to the entire enrolled population and to the unvaccinated) to assess predictors of immunity against HBV using sex, age, and nationality as predictors. The overall anti-HBs prevalence was 30%, with a significant decreasing trend in seropositivity with increasing age. The overall anti-HBc prevalence was 11.6%, with seropositivity increasing with age. Only one subject tested positive for HBsAg (0.2%). Approximately 67.4% (290/430) of the study population was susceptible, 20.9% (90/430) was vaccinated, 9.1% (39/430) had naturally acquired immunity, and 0.2% (1/430) had an acute infection. Older age and foreign nationality were identified as risk factors in both multivariate logistic regression models. The comparison highlights a reduction in the circulation of HBV infection markers (anti-HBc and HBsAg) over 30 years in Tuscany, particularly in younger age groups. Our seroprevalence study demonstrated a good level of protection against hepatitis B, primarily among individuals under 40 years old, the target group of the vaccination strategy.

## 1. Introduction

Hepatitis B is a liver disease caused by the hepatitis B virus (HBV), which can lead to both acute and chronic infection and represents a significant disease burden WHO estimates that 254 million people were living with chronic hepatitis B infection in 2022, with 1.2 million new infections each year; moreover, in the same year, hepatitis B resulted in an estimated 1.1 million deaths, mostly from cirrhosis and hepatocellular carcinoma (primary liver cancer) [1].

HBV is a DNA virus (*Hepadnaviridae* family) with three key antigens, “surface” (HBsAg), “core” (HBcAg), and “e” antigen (HBeAg), and it is transmitted through contact with infected bodily fluids, including blood, saliva, semen, and vaginal fluids [2]. The virus remains infectious on surfaces for up to seven days, facilitating transmission via contaminated needles, tattoos, and perinatal exposure [3]. HBV infection is often asymptomatic, particularly in infants and children. When symptomatic, it presents with jaundice, dark urine, fatigue, nausea, and abdominal pain. Acute infection typically lasts 1–3 months and may resolve following anti-HBs seroconversion. However, severe cases can result in fulminant liver failure and death [1,2]. Chronic HBV development is age-dependent: in fact, infection before age five leads to chronic disease in ~95% of cases, whereas fewer than 5% of adult infections become chronic [1].

Safe and effective vaccines against HBV exist and provide strong protection against acute and chronic HBV infection. Immunity persists for at least 20 years or is lifelong, with no need for booster doses [1]. However, immunization strategies vary by country. Italy was among the first countries to introduce HBV vaccination in 1991, mandating it for all newborns and 12-year-old adolescents. Since 2003, the vaccine has remained mandatory for infants, and in 2017, it became a school entry requirement for children aged 0–16 years [4,5]. The National Immunization Plan (NIP) schedules three doses at 3, 5, and 11 months, with an additional birth dose for infants born to HBsAg-positive mothers. High-risk groups such as healthcare workers, immunocompromised individuals, and injection drug users also receive vaccination [6]. WHO aims to eliminate hepatitis B by 2030 [7]. However, despite vaccination efforts, HBV prevalence remains uneven worldwide. The WHO African and Western Pacific Regions report the highest rates (7.5% and 5.9%), while Europe and the Americas show lower levels (1.5% and 0.5%) [8]. In 2021, 30 EU/EEA countries recorded 16,187 cases, mostly chronic [9]. In Italy, 89 acute cases (0.18 per 100,000) were reported (109 in 2022), primarily in males aged 35–54 years [10]. Tuscany recorded 20 cases (26 in 2022), 70% in men aged 35–64 years [11].

Seroepidemiological studies are useful tools for assessing the progress of implemented immunization strategies in achieving set goals. The aim of this study was to describe the seroprevalence of hepatitis B markers in a sample of the adult general population in the Province of Florence (Italy), based on sera collected between April 2018 and December 2019, and to contextualize these findings by comparing them with previous local surveys conducted 10 and 20 years after the implementation of the national HBV immunization strategy. We described the acquired immunity of the subjects enrolled according to the combination of three serological markers (anti-HBs, anti-HBc, and HBsAg).

## 2. Materials and Methods

This retrospective observational seroprevalence study belonged to a wider project named “Progetto Sierologia” carried out by the Department of Health Sciences of the University of Florence, in collaboration with Meyer Children’s Hospital and the Local Health Unit Toscana Centro in Italy. The protocol was approved by the local ethics committees (Project identification code: DSS-UNIFI, n. registro pareri 98/2017), and the study was conducted in accordance with the Declaration of Helsinki. The project aimed to assess the immunity/susceptibility levels towards the main vaccine-preventable infectious diseases in a representative sample of the pediatric and adult population residing in the Province of Florence [12,13,14,15,16,17,18].

### 2.1. Adult Enrollment, Sera Collection, and Sample Storage

The study population consisted of a convenience sample of adults aged 18–99 years residing in the Province of Florence, stratified by age and sex. The estimated sample size was 432 subjects, calculated as the sum of 0.1% of the resident population aged 18–19 years (16,743 individuals in 2015) and 0.05% of those aged ≥20 years (836,324 individuals). Enrollment took place at the Local Health Unit Presidio Morgagni (33, 50134 Florence) between April 2018 and December 2019. All participants provided written informed consent before blood sample collection. Subjects were excluded if they were non-residents of the Province of Florence, immunocompromised, receiving immunosuppressive treatment, had an acute infectious disease (measles, rubella, varicella, hepatitis A, or hepatitis B) in the two weeks before enrollment, or if they had received a blood transfusion within the previous six months. Blood samples were centrifuged (1600 rpm at 4 °C) for 10 min, and the recovered sera were stored at −20 °C until tested for hepatitis B markers. All sera were anonymized using a progressive numerical code.

### 2.2. Serological Analysis

All sera were tested for anti-HBs and total anti-HBc. Any sample that was anti-HBs negative and anti-HBc positive was subsequently tested for HBsAg. All tests were performed according to the manufacturer’s recommendations. A quantitative analysis was conducted to determine anti-HBs concentrations using the commercial Monolisa™ Anti-HBs Plus ELISA kit (Bio-Rad, Marnes-la-Coquette, France), which included five calibrators (C0: 0 mIU/mL; C1: 10 mIU/mL; C2: 100 mIU/mL; C3: 400 mIU/mL; C4: 1000 mIU/mL). Absorbance was measured at 450/630 nm and 405/630 nm. Sera with anti-HBs concentrations ≥10 mIU/mL were considered reactive and classified as seroprotected against HBV infection [1]. Samples with anti-HBs <10 mIU/mL were classified as negative. For qualitative detection of total anti-HBc (IgG and IgM), the Monolisa™ Anti-HBc PLUS ELISA kit (Bio-Rad, Marnes-la-Coquette, France) was used. The presence or absence of anti-HBc was determined by comparing sample absorbance to a cut-off value. Samples below the cut-off were considered negative, while those above or equal to the cut-off were initially classified as positive and required duplicate retesting with the same assay. A sample was confirmed positive if at least one of the two repeated tests was positive (≥cut-off); otherwise, it was classified as negative. Finally, samples with anti-HBs <10 mIU/mL but anti-HBc-positive were tested for HBsAg using the Monolisa™ HBsAg ULTRA direct ELISA (Bio-Rad, Marnes-la-Coquette, France), based on a calculated cut-off ratio.

(1) $$ratio\;\left(R\right)=\frac{sample\;absorbance}{cut-off\;value}$$

Samples with an *R* < 1 were considered as negative; samples with an *R* between 0.9 and 1 (0.9 < *R* < 1) were initially considered as equivocal, and the manufacturer suggested a re-test. Samples with *R* ≥ 1 were initially considered as positive and had to be re-tested in duplicate using the same assay (Monolisa™ HBsAg ULTRA) for the confirmation of the final result. Then, a sample was considered positive if at least one of the two duplicates had an *R* ≥ 1; otherwise, the sample was classified as negative.

### 2.3. Interpretation of the Acquired Immunity Towards Hepatitis B

The analyzed hepatitis B serological markers and their combinations were used to determine the immunity/susceptibility to HBV infection acquired by the enrolled subjects. We used the US Centers for Disease Control and Prevention (US CDC) guidelines for the interpretation of hepatitis B serologic test results (Table 1) [19].

### 2.4. Statistical Analysis

The serological results were collected in an Excel database and evaluated for anti-HBs antibody titers and the presence of anti-HBc and HBsAg. A descriptive analysis was performed to assess overall hepatitis B seroprevalence and its distribution according to the socio-demographic characteristics of the study population (sex, age, and nationality). The study population was divided into the following age groups: 18–29, 30–39, 40–49, 50–64, and >64 years. Subjects with dual nationality were classified as foreigners. To assess significant differences among the considered sociodemographic groups, the chi-square test and Fisher’s exact test were applied for comparisons between dichotomous variables (sex and nationality). Considering age groups as an ordinal variable, –Haenszel and Kendall’s tau-B tests were used to assess the presence of an increasing or decreasing linear trend between serological status (anti-HBs or anti-HBc) and age groups. Based on the US CDC’s recommendations for interpreting hepatitis B serological results, two dichotomous variables were established: “immune status” (susceptible vs. non-susceptible subjects) and “vaccination status” (immunized due to vaccination vs. all other profiles according to the US CDC). To investigate the relationship between HBV immunization status (acquired through vaccination or natural infection) and other variables such as sex, nationality, and age, a multivariate logistic regression analysis was performed. The Hosmer–Lemeshow test and area under the ROC curve were performed to assess the goodness of fit of the models. A *p*-value < 0.05 was considered statistically significant. All tests were performed using JAMOVI 2.3.21.0 [the jamovi project (2022), jamovi (Version 2.3) [Computer Software]; retrieved from https://www.jamovi.org].

## 3. Results

In the present study, 430 subjects aged 18 to 94 years were involved (mean age 51.8 ± 18.8 years). Female subjects represented 53.7% (N = 231), and men 46.3% (N = 199). The majority of the study population was composed of Italian subjects (87.4%; N = 376), while 12.6% (N = 54) were foreigners. More than half of the study population were over 50 years old (52.3%; N = 225) (Table 2).

### 3.1. Anti-HBs Seroprevalence in the Study Population

Considering anti-HBs antibodies, 70.0% (N = 301) of the subjects tested negative, while 30.0% (N = 129) screened positive (anti-HBs titers ≥ 10 mIU/mL). Positives were mainly aged 30–39 years (62.1%), male (30.7%), and Italian (91.2%) (Table 3). No significant differences were found based on nationality (*p* = 0.127) or sex (*p* = 0.784) for anti-HBs. With additional stratification by age, significant differences by nationality were observed in the 30–39-year-old and 50–64-year-old age groups. On the other hand, no differences were found based on sex in any age group (Supplementary Table S1). Trend tests (Mantel–Haenszel and Kendall’s tau-b) showed a significant trend (*p* < 0.001), with a decreasing probability of a positive anti-HBs serological status as age increased (Table 3).

### 3.2. Anti-HBc Seroprevalence in the Study Population

**Figure 1:**
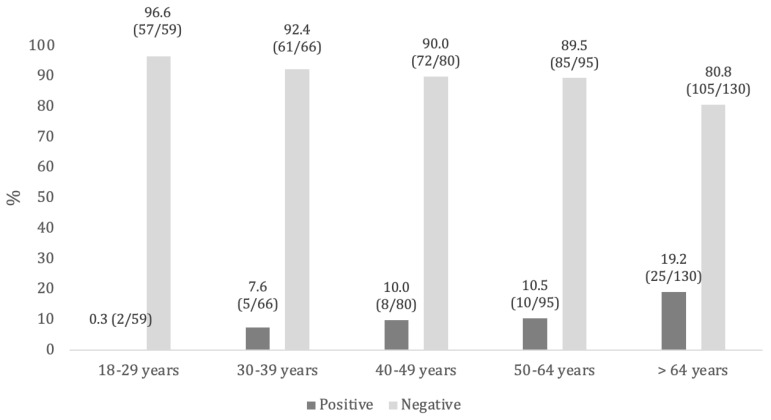
Anti-HBc seroprevalence, by age groups.

Considering anti-HBc antibodies, 88.4% (N = 380) of the subjects tested negative, while 11.6% (N = 50) screened positive. Positives were mainly aged over 64 years (19.2%), male (13.1%), and non-Italian (35.2%) (Table 4, Figure 1).

By evaluating the relationship between nationality and anti-HBc serological status, a statistically significant association was found for the entire study population and in certain age groups (18–29, 40–49, and 50–64 years). On the other hand, no significant differences were observed based on sex (Supplementary Table S2). Applying the Mantel–Haenszel and Kendall’s tau-B tests, we observed a significant increase (*p* < 0.001) in the probability of a positive anti-HBc serological status with increasing age (Kendall’s tau-B = 0.148; t = 3.40; Mantel–Haenszel = 11.1; gdl = 1) (Table 4).

### 3.3. Anti-HBs and Anti-HBc Pairing in the Study Population and HBsAg Seroprevalence

The analysis of pairing samples with an anti-HBc positivity and anti-HBs negativity (titers < 10 mIU/mL) identified 11 subjects, representing 2.6% of the study population (11/430). They were mainly aged >39 years, male, and with foreign nationality (Table 5).

The samples collected from these 11 subjects were tested for HBsAg presence. Among the 11 samples, only one resulted positive for the hepatitis B surface antigen and belonged to a Bangladeshi male aged 42 years old. The overall HBsAg prevalence in our sample was 0.2% (1/430).

### 3.4. Assessment of the Acquired Immunization

In agreement with the recommended interpretation of serological markers for hepatitis B from the US CDC, in the study population more than half of the subjects (67.4%, N = 290) were susceptible to hepatitis B virus infection. In contrast, 20.9% of subjects (N = 90) turned out to be vaccinated. A status compatible with a pattern of immunization from previous infection was found in 39 samples (9.1%). A very low percentage was calculated for the acute infection status (0.2%, 1/430) (Table 6).

The comparison between immunization due to HBV vaccination and all the other profiles according to the US CDC by sex and nationality showed no significant differences (*p*-value = 0.577 and *p*-value = 0.410, respectively).

**Figure 2:**
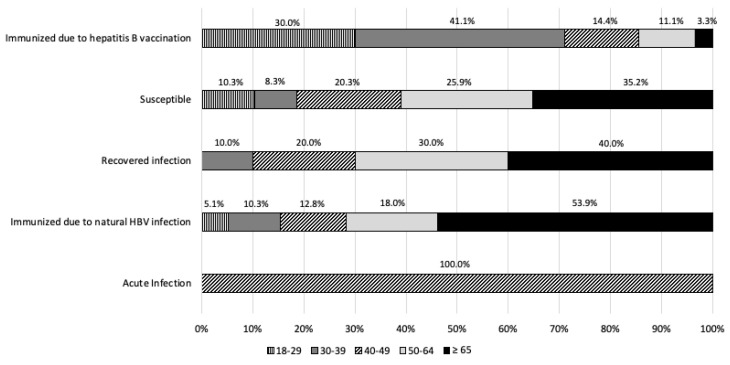
Interpretation of hepatitis B serological markers according to the US CDC, by age groups.

According to the age, susceptible subjects were mainly aged >39 years old; as a matter of fact, in the younger age groups, we found the higher percentage of immunization due to vaccination (45.8% in the 18–29-year-old age group and 56.0% in subjects aged 30–39 years old). Moreover, similar values were observed for natural infection-acquired immunity across different age groups, and the acute infection status was found only in the 40–49-year-old age group (0.2%) (Table 6, Figure 2).

By stratifying for age groups, no significant differences were found among those immunized due to HBV vaccination and all the other profiles, according to the US CDC. To assess the immunization status, a multivariate logistic regression analysis was performed. Subjects of foreign nationality were significantly more immunized against HBV (by vaccination or natural infection) than Italian individuals, even net of sex and age (OR = 2.178, *p* = 0.016). Differences between age groups were also observed. Specifically, the younger age group (18–29 years) was significantly more protected than all other age groups, except for the 30–39-year-old age group (OR = 1.756, *p* = 0.127). No association was found between immunity to HBV and sex (Table 7).

Performing the same test for the group of unvaccinated subjects (in this group the immune subjects are those who contracted the natural infection), it showed partially different results.

While the relationship between foreign nationality and immunity against hepatitis B infection was confirmed and became statistically stronger (OR 14.029, *p* < 0.001), results regarding differences between age groups in the study population were reversed. In this case, the group over 64 years appeared to have significantly higher immunity than all other age groups (Table 8).

## 4. Discussion

The aim of this study was to describe the seroprevalence of three main hepatitis B serological markers (anti-HBs, anti-HBc, and HBsAg) in the adult population residing in the Province of Florence and to assess the acquired immunity of the enrolled subjects according to the CDC’s interpretation for the three investigated serological markers. We found an overall anti-HBs prevalence of 30% and significant differences in anti-HBs seropositivity according to age. In fact, higher values were calculated in the youngest age groups: 49.2% in subjects aged 18–29 years and 62.1% in subjects aged 30–39 years. Moreover, the applied tests for trend confirmed a decrease in detectable anti-HBs with increasing age. Our findings align with Bonanni et al. [20] and Boccalini et al. [21] in confirming a certain decline of HBV infection markers in vaccinated cohorts, demonstrating the effectiveness of Italy’s universal vaccination program. However, compared to Boccalini et al. (2013) [20], which reported a 63% anti-HBs prevalence in those under 30, our study shows slightly lower levels (49.2% in 18–29 years), likely due to natural antibody waning without loss of immune memory. Unlike previous studies, our analysis also highlights the impact of nationality on HBV exposure, showing that foreign-born individuals are at higher risk of past infection.

Little recent data were available for anti-HBs seroprevalence in the Italian general population: a study carried out in Naples (Southern Italy) in 2015 found an overall anti-HBs seroprevalence of 16.1%, which dropped to 10.4% when anti-HBc was also concomitantly positive. In particular, anti-HBs seroprevalence was higher among subjects aged under 30 years (71.5%), which aligns with our findings [22]. The differences in anti-HBs prevalence by age groups may reflect immunization strategies implemented in Italy since 1991, which offered hepatitis B vaccination to all newborns and adolescents aged 12 years [4]. This means that subjects born from approximately 1980 onward have benefited from active vaccination, and it is reasonable to find higher anti-HBs prevalence in this population than in older age groups. Taking into account studies performed on high-risk groups such as students, trainees, or medical residents of different Italian universities (Bari, Chieti, and Siena) and healthcare workers (a teaching hospital in Rome), generally more than half of the study population showed positivity for anti-HBs, ranging from 54.0% to 85.7% in different surveys [23,24,25,26]. These higher seroprevalence values, compared to our results, highlight the strong recommendation for hepatitis B vaccination in these high-risk groups due to professional exposure [6]. Concerning the presence of infection serological markers, we found that 11.6% of the study population tested positive for anti-HBc, and only one subject was positive for HBsAg (0.2%). Significant differences were found in anti-HBc prevalence according to age and nationality. Indeed, anti-HBc prevalence significantly increased with age, ranging from 0.3% in subjects aged 18–29 years to 19.2% among those >64 years. Moreover, anti-HBc positivity was higher in foreign subjects compared to Italians (35.2% vs. 8.2%, respectively). Similar values were found among the resident population in Naples, with overall anti-HBc and HBsAg seroprevalence of 14.4% and 1.7%, respectively. Furthermore, those authors also observed an increase in anti-HBc prevalence with increasing age [23], consistent with our findings. These results highlight that the hepatitis B virus circulated more widely among the general population in past decades, and then—mainly thanks to the implementation of vaccination—its circulation has significantly decreased. As confirmed by data coming from the integrated epidemiological hepatitis system (SEIEVA), in Italy, hepatitis B incidence and the prevalence of infection markers have progressively decreased over the last 30 years [10,27,28]. In the period 1990–2019, the greatest decrease was recorded in the vaccination-targeted age groups, reaching a 100% reduction in new cases among the youngest population (0–14 years) and 99.4% in subjects aged 15–24 years [28]. Thus, our findings align with the Italian epidemiological trend. As regards nationality, in the last decade, Italy has been characterized by an increasing number of new hepatitis B cases, mainly due to sexual exposure with foreign subjects coming from countries endemic for hepatitis B [27].

From 2010 to 2019, about 20% of acute HBV cases occurred in foreign-born subjects [28]. A study on immigrants and refugees in Southern Italy highlighted high levels of seropositivity to hepatitis B markers: 9.6% tested positive for HBsAg and 40.4% for anti-HBc [29]. Therefore, foreigners—particularly those from countries with a high prevalence of hepatitis B—can be considered a fragile population that may facilitate virus circulation. In our study, the only HBsAg-positive participant was a 42-year-old man from Bangladesh, a country in Southeast Asia with an intermediate hepatitis B prevalence (3.0%).

Our analysis of acquired immunization showed that 67.4% of the enrolled population was susceptible (negative for all three serological markers), about 20.9% was immunized due to vaccination, and 9.1% was immunized due to natural infection. Considering the national recommendations for hepatitis B vaccination, we may have overestimated the proportion of susceptible individuals; in fact, in subjects who respond to a complete hepatitis B immunization cycle, anti-HBs titers naturally tend to decline over time and may become undetectable (<10 mIU/mL). As discussed in the literature, about 15–50% of children who respond to a primary three-dose immunization schedule have low or undetectable levels of anti-HBs 5–15 years post-vaccination [30,31]. Similarly, about 30–60% of subjects immunized in adulthood may have undetectable titers 9–11 years later [32]. Nevertheless, immune memory persists much longer, potentially for a lifetime [33]. Hence, the absence of circulating anti-HBs does not necessarily imply a lack of immunity in a vaccinated person, since immune memory remains effective [32]. For these reasons, no booster doses are needed for those who have completed the three-dose vaccination schedule [1,34].

We found that susceptible individuals were primarily older, whereas vaccinated individuals were mainly younger. Multivariate logistic regression of the entire study population showed that age is a predictor of acquired protection; specifically, younger age groups (18–29 and 30–39 years) are more protected than older groups. This correlates with the fact that cohorts up to 44 years of age were the target for the Italian anti-HBV vaccination at the time of our sampling. Moreover, our data show that nationality is also a predictor of acquired immunization, with foreign subjects being more immunized than Italians (OR = 2.178, *p* = 0.016). The multivariate logistic regression of the unvaccinated population confirmed that foreign nationality is significantly associated with naturally acquired immunity (OR = 14.029, *p* < 0.001) and that older age (>64 years) is a risk factor for having had a past HBV infection. Older age and being born abroad have been identified as risk factors for hepatitis B infection in other Italian studies [22,28].

This study presents several limitations that should be acknowledged. First, the retrospective design, relatively small sample size (430 subjects), and its restriction to a single geographic area (the Province of Florence) may limit the generalizability of the findings. Second, serum samples were collected over a limited timeframe (April 2018 to December 2019), which may not capture temporal fluctuations or longer-term trends in seroprevalence. Third, individual vaccination histories were not collected, preventing us from directly identifying vaccinated participants. As a result, our classification of immunization status relied solely on serological profiles, which may have led to an overestimation of the susceptible group, particularly among younger individuals with waning antibody levels. Moreover, while we tested for anti-HBs, anti-HBc, and HBsAg, we were unable to assess HBV DNA and therefore could not evaluate the possible presence of occult hepatitis B infection (OBI) among anti-HBc-only individuals. Finally, while we considered key sociodemographic variables (age, sex, nationality), other potentially relevant risk factors, such as socioeconomic status or previous healthcare exposure, were not assessed. Despite these limitations, our findings are consistent with previous national and regional data and provide relevant insights into the long-term impact of Italy’s hepatitis B vaccination strategy.

## 5. Conclusions

Our study confirms strong hepatitis B protection, especially in individuals under 40 years, demonstrating the success of Italy’s vaccination strategy. HBsAg prevalence has dropped to historically low levels compared to previous studies, highlighting reduced HBV circulation. To achieve elimination, reinforcing vaccination in high-risk groups and maintaining seroepidemiological surveillance are crucial. Public health efforts should focus on promoting vaccination in these groups, bringing Italy closer to hepatitis B elimination.

## Figures and Tables

**Table 1:** Interpretation of hepatitis B serological markers according to the US CDC (adapted from the US CDC).

Hepatitis B Serological Markers	Result	Interpretation
HBsAg	Negative	Susceptible to the HBV infection
Anti-HBc	Negative	
Anti-HBs	Negative	
HBsAg	Negative	Immunized due to natural HBV infection
Anti-HBc	Positive	
Anti-HBs	Positive	
HBsAg	Negative	Immunized due to hepatitis B vaccination
Anti-HBc	Negative	
Anti-HBs	Positive	
HBsAg	Positive	Acute infection if concomitant anti-HBc IgMs are positive. Chronic infection if concomitant anti-HBc IgMs are negative
Anti-HBc	Positive	
Anti-HBs	Negative	
HBsAg	Negative	Equivocal interpretation (4 possibilities): Recovered infection (most common) False-positive for anti-HBs, thus susceptible A low-level chronic infection Recovering from an acute infection
Anti-HBc	Positive	
Anti-HBs	Negative	

**Table 2:** Demographic characteristics (age, nationality, and sex) of the study population, by age groups.

Age Groups (Years)	Nationality (N)		Sex (N)		Enrolled Subject (N)
	Italian	Non-Italian	Male	Female	
18–29	50	9	29	30	59
30–39	52	14	32	34	66
40–49	64	16	37	43	80
50–64	83	12	46	49	95
>64	127	3	55	75	130
Total	376	54	199	231	430

**Table 3:** Anti-HBs seroprevalence in the study population divided by age groups, sex, and nationality.

	Anti-HBs Seroprevalence			
Variable	Group	Positive % (n/N)	Negative % (n/N)	*p*-Value *
Age groups (years)	18–29	49.2 (29/59)	51.8 (30/59)	<0.001
	30–39	62.1 (41/66)	37.9 (25/66)	
	40–49	22.5 (18/80)	77.5 (62/80)	
	50–64	17.9 (17/95)	82.1 (78/95)	
	>64	18.5 (24/130)	81.5 (106/130)	
Sex	Female	29.4 (68/231)	70.6 (163/231)	0.784
	Male	30.7 (61/199)	69.3 (138/199)	
Nationality	Italian	91.9 (108/376)	8.1 (268/376)	0.127
	Non-Italian	84.9 (21/54)	15.1 (33/54)	
Total		30.0 (129/430)	70.0 (301/430)	

Notes: *: Age groups: Kendall tau-b = −0.267; t = 3.40. Mantel–Haenszel = 40.7; gdl = 1. Sex and nationality: Chi-square.

**Table 4:** Anti-HBc seroprevalence in the study population divided by age groups, sex, and nationality.

		Anti-HBc Seroprevalence		
Variable	Group	Positive % (n/N)	Negative % (n/N)	*p*-Value *
Age (years)	18–29	0.3 (2/59)	96.6 (57/59)	
	30–39	7.6 (5/66)	92.4 (61/66)	
	40–49	10.0 (8/80)	90.0 (72/80)	<0.001
	50–64	10.5 (10/95)	89.5 (85/95)	
	>64	19.2 (25/130)	80.8 (105/130)	
Sex	Female	10.4 (24/231)	89.6 (207/231)	0.338
	Male	13.1 (26/199)	86.9 (173/199)	
Nationality	Italian	8.2 (31/376)	91.7 (345/376)	<0.001
	Non-Italian	35.2 (19/54)	64.8 (35/54)	
Total		11.6 (50/430)	88.4 (380/430)	

Notes: *: Age groups: Kendall tau-b = −0.148; t= 3.40. Mantel–Haenszel = 11.1; gdl = 1. Sex and nationality: Chi-square.

**Table 5:** Sociodemographic characteristics of subjects who tested negative for anti-HBs (titers < 10 mIU/mL) and positive for anti-HBc.

		Anti-HBs Negative—Anti-HBc Positive Pairing
Variable	Group	% (n/N)
Age (years)	18–29	0.0 (0/59)
	30–39	1.5 (1/66)
	40–49	3.8 (3/80)
	50–64	3.2 (3/95)
	>64	3.1 (4/130)
Sex	Female	0.9 (2/231)
	Male	4.5 (9/199)
Nationality	Italian	1.1 (4/376)
	Non-Italian	13.0 (7/54)
Total		2.6 (11/430)

**Table 6:** Demographic characteristics (nationality and sex) of the study population, by interpretation of hepatitis B serological markers according to the US CDC.

Status (CDC)	Nationality (N)		Sex (N)		Enrolled Subjects% (N)
	Italian	Non-Italian	Male	Female	
Acute Infection	0	1	1	0	0.2 (1/430)
Immunized due to natural HBV infection	27	12	17	22	9.1 (39/430)
Resolved infection or other possibilities	4	6	8	2	2.3 (10/430)
Susceptible	264	26	129	161	67.4 (290/430)
Immunized due to hepatitis B vaccination	81	9	46	44	20.9 (90/430)
Total	376	54	201	229	100.0 (430/430)

**Table 7:** Multivariate logistic regression analysis: probability of being immunized against HBV in the overall study population (Hosmer–Lemeshow = 6.96, *p* = 0.2237, area under ROC curve = 0.7120).

Dependent Variable: Immunization Status (Probability of Being Immunized Against HBV)					
		OR	SE	CI 95%	*p*-Value
Age groups (years)	18–29	-	-	-	-
	30–39	1.756	0.369	0.851–3.621	0.127
	40–49	0.348	0.370	0.168–0.718	0.004
	50–64	0.275	0.366	0.134–0.563	<0.001
	>64	0.316	0.341	0.162–0.617	<0.001
Sex	Female	-	-	-	-
	Male	1.241	0.222	0.804–1.916	0.330
Nationality	Italian	-	-	-	-
	Non-Italian	2.178	0.324	1.155–4.106	0.016

Note: OR: odds ratio; SE: standard error; CI: confidence interval; “-”: reference.

**Table 8:** Multivariate logistic regression analysis for unvaccinated subjects (Hosmer–Lemeshow = 2.89, *p* = 0.7166, area under ROC curve = 0.7681).

Dependent Variable: Immunization Status for Natural Infection					
		OR	SE	CI 95%	*p*-Value
Age groups (years)	18–29	0.100	0.882	0.018–0.564	0.009
	30–39	0.202	0.685	0.053–0.774	0.020
	40–49	0.207	0.548	0.071–0.606	0.004
	50–64	0.274	0.477	0.108–0.699	0.007
	>64	-	-	-	-
Sex	Female	-	-	-	-
	Male	1.517	0.417	0.788–2.922	0.212
Nationality	Italian	-	-	-	-
	Non-Italian	14.029	0.466	5.624–34.995	<0.001

Note: OR: odds ratio; SE: standard error; CI: confidence interval; “-”: reference.

## Data Availability

The original contributions presented in this study are included in the article/Supplementary Materials. Further inquiries can be directed to the corresponding authors.

## Supplementary Materials

The following supporting information can be downloaded at: https://www.mdpi.com/article/10.3390/pathogens14040362/s1, Table S1: Differences on the basis of nationality and sex for anti-HBs by age groups. Table S2: Differences on the basis of nationality and sex for anti-HBc by age groups.

## References

[1] World Health Organization (WHO) Hepatitis B. https://www.who.int/news-room/fact-sheets/detail/hepatitis-b.

[2] Haber P., Schillie S., Hall E., Wodi A.P., Hamborsky J., Morelli V., Schillie S. (2021). Chapter 10: Hepatitis B. Epidemiology and Prevention of Vaccine-Preventable Diseases.

[3] Bond W.W., Favero M.S., Petersen N.J., Gravelle C.R., Ebert J.W., Maynard J.E. (1981). Survival of hepatitis B virus after drying and storage for one week. Lancet.

[4] Gazzetta Ufficiale Legge n. 165 del 27 Maggio 1991. Obbligatorietà della Vaccinazione contro L’epatite Virale B. https://www.gazzettaufficiale.it/atto/serie_generale/caricaDettaglioAtto/originario?atto.dataPubblicazioneGazzetta=1991-06-01&atto.codiceRedazionale=091G0201&elenco30giorni=false.

[5] Gazzetta Ufficiale Legge n. 119 del 31 Luglio 2017. Conversione in Legge, con Modificazioni, del Decreto-Legge 7 Giugno 2017, n. 73, Recante Disposizioni Urgenti in Materia di Prevenzione Vaccinale. https://www.gazzettaufficiale.it/atto/serie_generale/caricaDettaglioAtto/originario?atto.dataPubblicazioneGazzetta=2017-08-05&atto.codiceRedazionale=17G00132&elenco30giorni=false.

[6] Ministero della Salute Vaccinazioni. Piano Nazionale Prevenzione Vaccinale 2023–2025. https://www.salute.gov.it/portale/vaccinazioni/dettaglioContenutiVaccinazioni.jsp?lingua=italiano&id=4828&area=vaccinazioni&menu=vuoto.

[7] World Health Organization (WHO) Combating Hepatitis B and C to Reach Elimination by 2030. https://apps.who.int/iris/bitstream/handle/10665/206453/WHO_HIV_2016.04_eng.pdf.

[8] World Health Organization (WHO) (2022). World Health Statistics 2022: Monitoring Health for the SDGs, Sustainable Development Goals.

[9] European Centre for Disease Prevention and Control (ECDC) Hepatitis B. https://www.ecdc.europa.eu/en/hepatitis-b.

[10] Istituto Superiore di Sanità (ISS) SEIEVA. Bollettino SEIEVA Epidemiologia Delle Epatiti Virali Acute In Italia. Numero 12. Aggiornamento 2022—Marzo 2023. https://www.epicentro.iss.it/epatite/Bollettino-Seieva.

[11] Agenzia Regionale di Sanità della Toscana (ARS) Sorveglianza epidemiologica delle malattie infettive in Toscana 2022. https://www.ars.toscana.it/images/pubblicazioni/Rapporti/2023/Rapporto_malattie_infettive_2022_def.pdf.

[12] Bechini A., Zanella B., Bonito B., Betti M., Stancanelli E., Del Riccio M., Salvati C., Bonanni P., Bianchi J., Biondi I. (2024). Anti-rubella seroprevalence assessment in an adult sample population in Italy. Ann. Ig. Med. Prev. E Comunità.

[13] Bechini A., Del Riccio M., Salvati C., Bonito B., Zanella B., Biamonte M.A., Bruschi M., Iamarino J.A., Fattorini L., Baggiani L. (2024). Seroprevalence Assessment of Anti-Varicella Antibodies among Adults in the Province of Florence (Italy). Vaccines.

[14] Zanella B., Bechini A., Bonito B., Del Riccio M., Ninci A., Tiscione E., Bonanni P., Working Group DHS, Working Group AOUMeyer, Working Group AUSLTC (2021). A Study of Varicella Seroprevalence in a Pediatric and Adolescent Population in Florence (Italy). Natural Infection and Vaccination-Acquired Immunization. Vaccines.

[15] Zanella B., Boccalini S., Biamonte M.A., Giorgetti D., Menicacci M., Bonito B., Ninci A., Tiscione E., Puggelli F., Mereu G. (2021). A Study of Hepatitis A Seroprevalence in a Paediatric and Adolescent Population of the Province of Florence (Italy) in the Period 2017–2018 Confirms Tuscany a Low Endemic Area. Vaccines.

[16] Zanella B., Boccalini S., Bonito B., Del Riccio M., Manzi F., Tiscione E., Bonanni P., Working Group DHS, Working Group AOUMeyer, Working Group AUSLTC (2020). Rubella Seroprevalence Boost in the Pediatric and Adolescent Population of Florence (Italy) as a Preventive Strategy for Congenital Rubella Syndrome (CRS). Vaccines.

[17] Zanella B., Boccalini S., Bonito B., Del Riccio M., Tiscione E., Bonanni P., Bechini A., Working Group DHS, Working Group AOUMeyer, Working Group AUSLTC (2020). Increasing Measles Seroprevalence in a Sample of Pediatric and Adolescent Population of Tuscany (Italy): A Vaccination Campaign Success. Vaccines.

[18] Zanella B., Bechini A., Boccalini S., Sartor G., Tiscione E., Bonanni P., Working Group DHS, Working Group AOUMeyer, Working Group AUSLTC (2020). Hepatitis B Seroprevalence in the Pediatric and Adolescent Population of Florence (Italy): An Update 27 Years after the Implementation of Universal Vaccination. Vaccines.

[19] Centers for Disease Control and Prevention Division of Viral Hepatitis. Interpretation of Hepatitis B Serologic Test Results. https://www.cdc.gov/hepatitis-b/hcp/diagnosis-testing/?CDC_AAref_Val=https://www.cdc.gov/hepatitis/hbv/interpretationOfHepBSerologicResults.htm.

[20] Boccalini S., Pellegrino E., Tiscione E., Pesavento G., Bechini A., Levi M., Rapi S., Mercurio S., Mannelli F., Peruzzi M. (2013). Sero-epidemiology of hepatitis B markers in the population of Tuscany, Central Italy, 20 years after the implementation of universal vaccination. Hum. Vaccines Immunother..

[21] Bonanni P., Pesavento G., Bechini A., Tiscione E., Mannelli F., Benucci C., Nostro A.L. (2003). Impact of universal vaccination programmes on the epidemiology of hepatitis B: 10 years of experience in Italy. Vaccine.

[22] Morisco F., Stroffolini T., Lombardo F.L., Guarino M., Camera S., Cossiga V., Donnarumma L., Loperto I., Caporaso N. (2017). Prevalence of and risk factors for HBV infection in a metropolitan Southern Italian area: Evidence for the effectiveness of universal Hepatitis B vaccination. Dig. Liver Dis..

[23] Bianchi F.P., Gallone M.S., Gallone M.F., Larocca A.M., Vimercati L., Quarto M., Tafuri S. (2019). HBV seroprevalence after 25 years of universal mass vaccination and management of non-responders to the anti-Hepatitis B vaccine: An Italian study among medical students. J. Viral Hepat..

[24] Di Giampaolo L., Costantini E., Di Nicola M., Porreca A., D’Amore G., Coppeta L., Mangifesta R. (2022). Titer of anti-HBs in health professions trainees: Prevalence of antibody coverage in a University of Central Italy. Hum. Vaccines Immunother..

[25] Sartorelli P., Occhialini F., Miceli R., Pietronigro A., Bianciardi L., Salini C., Messina G. (2022). The seroprevalence of the hepatitis B virus in Italian medical students after three decades since the introduction of universal vaccination. Int. J. Occup. Med. Environ. Health.

[26] Coppeta L., D’Alessandro I., Pietroiusti A., Somma G., Balbi O., Iannuzzi I., Magrini A. (2021). Seroprevalence for vaccine-preventable diseases among Italian healthcare workers. Hum. Vaccines Immunother..

[27] Ministero della Salute (2015). Piano Nazionale per la Prevenzione delle Epatiti Virali da Virus B e C (PNEV). https://www.salute.gov.it/imgs/C_17_pubblicazioni_2437_allegato.pdf.

[28] Stroffolini T., Morisco F., Ferrigno L., Pontillo G., Iantosca G., Cossiga V., Crateri S., Tosti M.E., The SEIEVA Collaborating Group (2022). Effectiveness of Hepatitis B Vaccination Campaign in Italy: Towards the Control of HBV Infection for the First Time in a European Country. Viruses.

[29] Coppola N., Alessio L., Gualdieri L., Pisaturo M., Sagnelli C., Minichini C., Di Caprio G., Starace M., Onorato L., Signoriello G. (2017). Hepatitis B virus infection in undocumented immigrants and refugees in Southern Italy: Demographic, virological, and clinical features. Infect. Dis. Poverty.

[30] Assad S., Francis A. (1999). Over a decade of experience with a yeast recombinant hepatitis B vaccine. Vaccine.

[31] Van Damme P., Ward J.W., Shouval D., Zanetti A., Plotkin S.A., Orenstein W., Offit P.A., Edwards K.M. (2018). Hepatitis B Vaccines. Plotkin’s Vaccines.

[32] Leuridan E., Van Damme P. (2011). Hepatitis B and the need for a booster dose. Clin. Infect. Dis..

[33] Banatvala J.E., Van Damme P. (2003). Hepatitis B vaccine—Do we need boosters?. J. Viral Hepat..

[34] Bruce M.G., Bruden D., Hurlburt D., Zanis C., Thompson G., Rea L., Toomey M., Townshend-Bulson L., Rudolph K., Bulkow L. (2016). Antibody levels and protection after hepatitis B vaccine: Results of a 30-year follow-up study and response to a booster dose. J. Infect. Dis..

