# Supplementary material for: Impact of the Hepatitis B Immunization Strategy Adopted in Italy from 1991: The Results of a Seroprevalence Study on the Adult Population of Florence, Italy
Source: Pathogens. 2025 Apr 7;14(4):362. doi: 10.3390/pathogens14040362 (PMC12030553; doi:10.3390/pathogens14040362)
Supplement: Supplementary file 1 [file pathogens-14-00362-s001.zip › pathogens-3536544-supplementary.pdf]

Supplementary Materials

Table S1. Differences on the basis of nationality and sex for anti-HBs by age groups.

		Age groups											
		18-29		30-39		40-49		50-64		>64		Total	
		**Positive**	**Negative**	**Positive**	**Negative**	**Positive**	**Negative**	**Positive**	**Negative**	**Positive**	**Negative**	**Positive**	**Negative**
**Nationality (N)**	**Italian**	23	27	36	16	13	51	12	71	24	103	108	268
	**Non-Italian**	6	3	5	9	5	11	5	7	0	3	21	33
	**Fisher's exact test**	**Value**	***p***	**Value**	***p***	**Value**	***p***	**Value**	***p***	**Value**	***p***	**Value**	***p***
		1.303	0.299	5.266	0.031	0.878	0.338	5.283	0.037	0.695	1.000	2.324	0.152
**Sex (N)**	**Female**	13	17	22	12	10	33	10	39	13	62	163	68
	**Male**	16	13	19	13	8	29	7	39	11	44	138	61
	**Fisher's exact test**	**Value**	***p***	**Value**	***p***	**Value**	***p***	**Value**	***p***	**Value**	***p***	**Value**	***p***
		0.8270	0.439	0.1991	0.800	0.0305	1.000	0.4351	0.597	0.1499	0.820	0.0753	0.833

Table S2. Differences on the basis of nationality and sex for anti-HBc by age groups.

		Age groups											
		18-29		30-39		40-49		50-64		>64		Total	
		**Positive**	**Negative**	**Positive**	**Negative**	**Positive**	**Negative**	**Positive**	**Negative**	**Positive**	**Negative**	**Positive**	**Negative**
**Nationality (N)**	**Italian**	50	7	50	11	62	10	80	5	103	2	345	35
	**Non-Italian**	0	2	2	3	2	6	3	7	24	1	31	19
	**Fisher's exact test**	**Value**	**p**	**Value**	**p**	**Value**	**p**	**Value**	**p**	**Value**	**p**	**Value**	**p**
		11.501	0.021	4.870	0.060	16.806	<0.001	33.330	< 0.001	0.393	0.476	33.351	<0.001
**Sex (N)**	**Female**	0	30	4	30	3	40	5	44	12	63	24	207
	**Male**	2	27	1	31	5	32	5	41	13	42	26	173
	**Fisher's exact test**	**Value**	**p**	**Value**	**p**	**Value**	**p**	**Value**	**p**	**Value**	**p**	**Value**	**p**
		21.416	0.237	17.574	0.357	0.9442	0.461	0.0112	1.000	11.913	0.368	0.7448	0.451
